# Observation of finite-wavelength screening in high-energy-density matter

**DOI:** 10.1038/ncomms7839

**Published:** 2015-04-23

**Authors:** D. A. Chapman, J. Vorberger, L. B. Fletcher, R. A. Baggott, L. Divol, T. Döppner, R. W. Falcone, S. H. Glenzer, G. Gregori, T. M. Guymer, A. L. Kritcher, O. L. Landen, T. Ma, A. E. Pak, D. O. Gericke

**Affiliations:** 1 AWE plc, Radiation Physics Department, Aldermaston, Reading RG7 4PR, UK; 2 Centre for Fusion, Space and Astrophysics, Department of Physics, University of Warwick, Coventry CV4 7AL, UK; 3 Max-Planck-Institut für die Physik komplexer Systeme, Dresden 01187, Germany; 4 High-Energy-Density Science Division, SLAC National Accelerator Laboratory, Menlo Park, California 94025, USA; 5 National Ignition Facility and Photon Science Directorate, Lawrence Livermore National Laboratory, Livermore, California 94550, USA; 6 Physics Department, University of California, Berkeley, California 94720, USA; 7 Department of Physics, University of Oxford, Parks Road, Oxford OX1 3PU, UK

## Abstract

A key component for the description of charged particle systems is the screening of the Coulomb interaction between charge carriers. First investigated in the 1920s by Debye and Hückel for electrolytes, charge screening is important for determining the structural and transport properties of matter as diverse as astrophysical and laboratory plasmas, nuclear matter such as quark-gluon plasmas, electrons in solids, planetary cores and charged macromolecules. For systems with negligible dynamics, screening is still mostly described using a Debye–Hückel-type approach. Here, we report the novel observation of a significant departure from the Debye–Hückel-type model in high-energy-density matter by probing laser-driven, shock-compressed plastic with high-energy X-rays. We use spectrally resolved X-ray scattering in a geometry that enables direct investigation of the screening cloud, and demonstrate that the observed elastic scattering amplitude is only well described within a more general approach.

## Abstract (web summary)

Charge screening dominates the behaviour of high-energy plasmas, which exist in stars and possibly in future fusion technology. Here, the authors describe a theoretical framework for charge screening that goes beyond the conventional model and demonstrate its importance in analysing experimental data.

## Graphical abstract

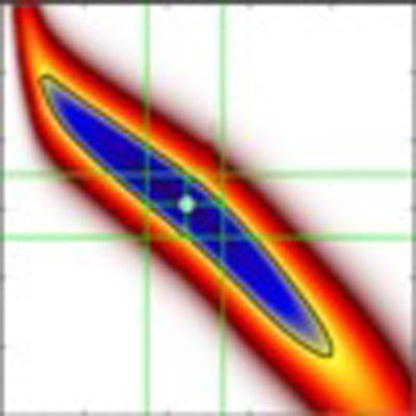

The Debye–Hückel theory of charge screening^1^ is one of the seminal results of electrolyte and plasma physics, whereupon the long-range Coulomb force between a pair of charge carriers is replaced by an exponentially decaying, short-range potential to account for the interaction with the surrounding medium. The extension of this classical description to degenerate electrons, Thomas–Fermi screening^2^,^3^, plays an important role in the description of electrons in solids^4^ and warm dense matter (WDM)^5^. Moreover, the concept of Debye-like screening is also applied to describe a large range of systems usually not associated with particles interacting via Coulomb forces, including quark-gluon matter^6^,^7^, ultra-cold systems in traps^8^ or chemical and biological systems^9^. For all these cases, the structural, thermodynamic, transport or relaxation properties are determined by effective interactions between localized charges.

Although Debye-like screening has been applied for many applications, it contains a number of inherent restrictions: weakly interacting particles in the screening cloud, negligible dynamic evolution and the long-wavelength limit. The latter requirement is only fulfilled for processes with a wavelength much larger than the screening length, leading to a wavenumber *k* effectively approaching zero. In contrast, we will refer to static screening models, which are not similarly restricted, as finite-wavelength screening.

To investigate static screening beyond the long-wavelength limit, processes involving large momentum transfers need to be considered. Large-angle collisions are a typical process where finite-wavelength screening could be observed. Although such collisions are highly unlikely in ideal, low-density plasmas, strong scattering is known to modify transport and relaxation properties in dense plasmas^10^,^11^. Another possibility to investigate deviations from Debye-like screening is the interaction of X-rays with dense matter under large scattering angles^12^. Indeed, spectrally resolved X-ray Thomson scattering (XRTS)^13^ is particularly suited for these investigations, as it simultaneously allows for the determination of the plasma conditions and the study of the screening cloud from a single spectrum.

In the following, we report observations of finite-wavelength screening in dense matter probed via spectrally resolved XRTS on laser-driven, shock-compressed plastic (CH) capsules. The strength of the elastic Rayleigh feature is used to further constrain simultaneous measurements of the electron density, temperature and mean ionization obtained from the inelastic Compton feature. We show that agreement between modelled and measured values for the Rayleigh amplitude can only be obtained if finite-wavelength screening is considered.

## Results

### X-ray scattering in high-energy-density matter

Investigation of the screening cloud at large wavenumbers requires highly compressed and moderately heated matter to be probed by high-energy X-rays. These target conditions were created at the Omega laser facility by compressing thin CH shells with multiple coalescent shocks driven by intense laser beams. He-α radiation at ∼9 keV from a laser-produced Zn plasma is used as the probe. Measuring the intensity of the X-ray scatter for angles between 120° and 150° accesses the response at the wavenumbers of interest (see Methods).

The spectrum of radiation scattered by a plasma is proportional to the double-differential scattering cross-section^14^


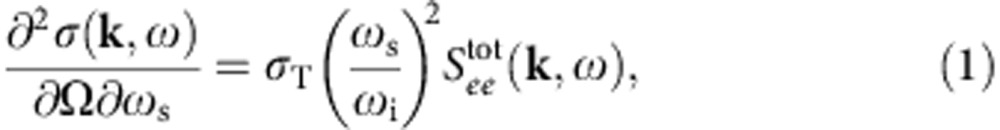


where *σ*_T_=6.65 × 10^−25^ cm^2^ is the Thomson cross-section. The frequency and wavenumber shifts associated with the scattering are *ω*=*ω*_i_–*ω*_s_ and **k**=**k**_i_–**k**_s_, respectively. 
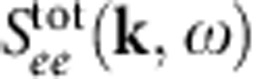
 is the total dynamic structure factor containing information on the spatio-temporal correlations of the fully coupled electron system. For a composite plasma probed with high-energy X-rays, the latter can be decomposed into distinct contributions from elastic and inelastic scattering^15^. Both terms depend on the temperature, density and mean charge state of the ions. Thus, in principle, the thermodynamic state of the target may be determined by fitting the measured spectrum with theoretical calculations^16^,^17^,^18^.

It is well known that robust estimates of the electron density and temperature of the samples probed with XRTS can be made by matching to the inelastic Compton scattering feature of the spectrum^19^,^20^. Moreover, the relative contributions of free and bound electrons enable the ionization state, and therefore material density, to be measured^21^. Uncertainties in the models describing this feature are effectively constrained by probing at large *k* (see Methods). In this work, we obtain conditions suggestive of a WDM state with free-electron densities of ∼10^24^ cm^−3^ and temperatures of 
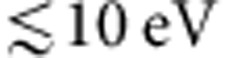
. Furthermore, we find the mean charge state of the carbon ions to be 
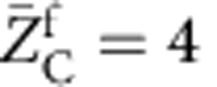
, consistent with estimates of continuum lowering in WDM.

In contrast, the elastic Rayleigh feature is the result of scattering from electrons that follow the low-frequency density fluctuations of the ions and, thus, is an excellent measure for the static ionic structure and the associated screening properties^22^,^23^,^24^,^25^. The elastic scattering strength in WDM with multiple ion species is given by^26^


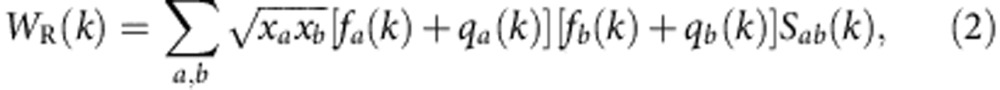


where 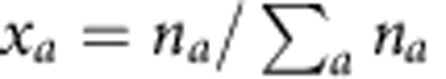 is the concentration of ion species *a*, with *n*_*a*_ its density, *f*_*a*_(*k*) is the form factor of bound electrons and *q*_*a*_(*k*) is the screening cloud^13^. The latter quantities are Fourier transforms of the respective electron densities around ions of species *a*. *S*_*ab*_(*k*) denotes the partial ion–ion structure factors. The form factors for bound electrons are readily obtained from first-principles methods for the tightly bound K-shell electrons in carbon.

**Figure 1:**
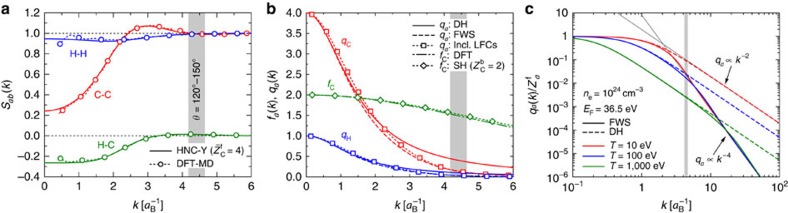
Elastic scattering contributions and limiting behaviour of the screening cloud. (**a**) Partial ion–ion structure factors *S*_*ab*_(*k*) for CH with 
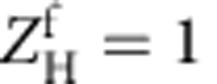
, 
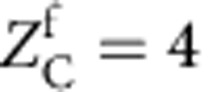
 at *ρ*=5.84 g cm^−3^ (*n*_*e*_=1.3 × 10^24^ cm^−3^) and *T*=10 eV applying multicomponent classical hypernetted-chain calculations (HNC—solid curves) with screened Coulomb interactions between ions, and density functional molecular dynamics (DFT-MD—dotted curves with circles). (**b**) Screening clouds *q*_*a*_(*k*) around the H and C ions for the same conditions as **a** calculated with Debye-like (DH—solid curves) and finite-wavelength screening (FWS—dashed curves) approaches. Corrections to the RPA due to strong static correlations (dotted curve with squares) are negligible for both components at large *k*. The form factors of the bound electrons around the C ions based on the density functional theory (DFT—dot-dashed curve) and screened hydrogenic wavefunctions (SH—dotted curve with diamonds) are also shown for comparison. (**c**) Behaviour of the charge-normalized screening cloud 
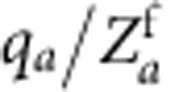
 for various temperatures at *n*_*e*_=10^24^ cm^−3^, comparing the FWS (solid curves) and DH (dashed curves) models. The different scaling in the limits *k*→0 and *k*→∞ are demonstrated with thin grey lines. The range of wavenumbers probed by in this experiment is shown by the vertical shaded band in all panels.

For most conditions, the largest theoretical uncertainty in *W*_R_ is related to the ionic structure factors (see, for example, ref. 27). In the present experiment, the ambiguity with respect to the ionic structure is circumvented by the large scattering angle applied as shown in Fig. 1a. At the resulting large *k* values (shaded vertical band), all spatial correlations have decayed and we have unity for *S*_*aa*_ and negligible values for *S*_*ab*_. This result is independent of the method applied to obtain the ion structure (see Methods). Thus, the only unknown remaining in the description of the elastic scattering strength is the form of the screening cloud *q*_*a*_(*k*), which can be determined by our measurements.

### Theoretical description of the screening cloud

In general, the response of the electrons to the ions may be derived within a quantum statistical framework from the ratio of dynamic structure factors, thereby rigorously incorporating strong interactions. As it is associated with the ions, the frequency dependence of the screening cloud is negligible since the electrons react to the ion distribution almost instantaneously, that is, electronic screening can be treated in the static limit *ω*→0. For the conditions of interest, electron–ion correlations are expected to be weak by virtue of the high Fermi energy, justifying a linear response approach^28^


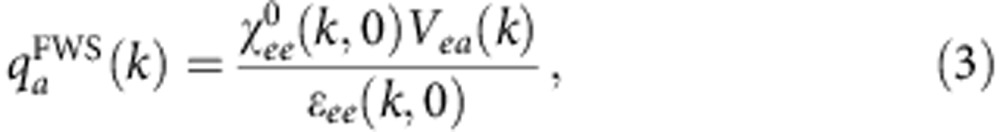


in which the screening is provided by the dielectric function 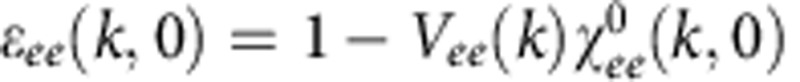. Strong correlations between electrons can be accounted for by modifying the potential with local field corrections 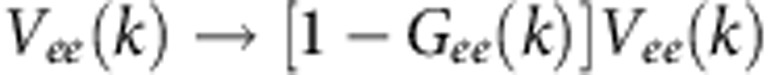. However, a weak coupling approximation, *G*_*ee*_(*k*)≈0, may be assumed for the conditions created. The non-interacting density response is then given by the random phase approximation (RPA)^5^


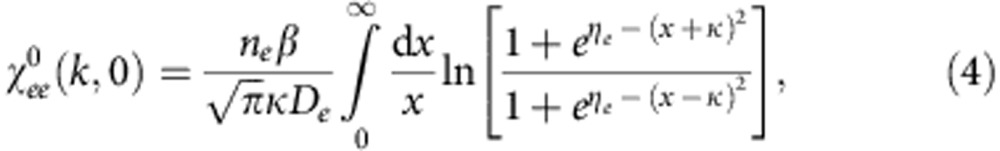


where we have defined 
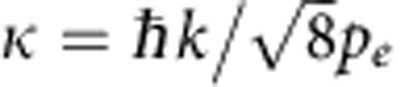
, 
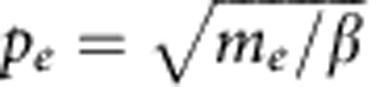
 and *β*=1/*k*_B_*T*. Furthermore, 
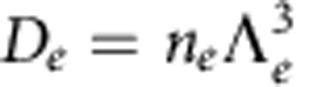
 is the degeneracy parameter, with 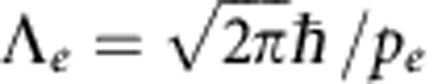 being the thermal de Broglie wavelength, and *η*_*e*_=*βμ*_*e*_ is the dimensionless chemical potential. Note that equation (4) is valid for arbitrary *k*, making it suitable to describe finite-wavelength screening.

While the idea of such a generalized approach to screening is not new, until recently the vast majority of XRTS data has been evaluated on the basis of the Debye–Hückel or Thomas–Fermi theories, wherein the dielectric function has the form 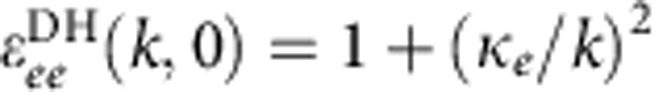. The inverse screening length *κ*_*e*_ is chosen to be consistent with the appropriate limit, but may also be derived for arbitrary degeneracies^5^. One may readily obtain this form of the dielectric function by taking the long-wavelength (*k*→0) limit of equation (4). Thus, the Debye-like (Debye–Hückel and Thomas–Fermi) theories are restricted to small momentum transfer events, such as long-range interactions between particles.

Taking the electron–ion interaction to be the Coulomb potential, one may assume 
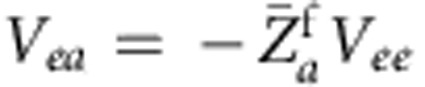
 and subsequently find the well-known expression for the screening cloud


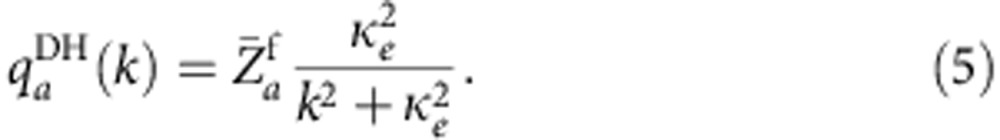


Reviewing equation (5) reveals that the ratio *k*/*κ*_*e*_ determines whether the long-wavelength limit is applicable. For collective scattering^29^,^30^, the wavelength of density fluctuations is larger than the screening length: *α*=*κ*_*e*_/*k*>1. Accordingly, the long-wavelength limit is valid. Conversely, we have *α*<1 in the non-collective scattering regime, and the screening function arising from combining equations (3) and (4) should be used. In this regime, the tail of the screening cloud decays significantly faster than the 1/*k*^2^-scaling predicted by the Debye-like theories equation (5) as can be seen in Fig. 1b. Under non-degenerate conditions, however, the Debye-like behaviour is recovered for intermediate *k* before changing to scale as 1/*k*^4^ in the limit *k*→∞ (Fig. 1c). Note that the finite-wavelength results tend to a constant for large *k* as the electron density close to the ions becomes independent of the temperature due to quantum degeneracy.

Furthermore, this strong 1/*k*^4^ decay directly leads to the well-known Friedel oscillations of the real-space electron density distribution around the ions^31^, which have been observed in cold solids^32^. This effect has thus far been hidden in XRTS experiments by probing higher temperatures, use of smaller scattering angles or weak ionization. In our experiments, the large scattering angle combined with strongly driven WDM states results in an intermediate scattering regime with *α*∼0.3, where the screening cloud exhibits changes due to finite-wavelength effects and also gives a significant contribution to the elastic Rayleigh feature.

### Data analysis

To determine the plasma conditions from the measured XRTS spectra, we apply a statistical analysis with respect to the probability density corresponding to *χ*^2^ statistics (see, for example, ref. 33) over a wide range of density–temperature space. The best fit is defined by minimizing the mean square deviation between data and model, that is *χ*^2^, whereas the quality and robustness of the fit is estimated by the values of *χ*^2^ in the density–temperature region around the best fit.

**Figure 2:**
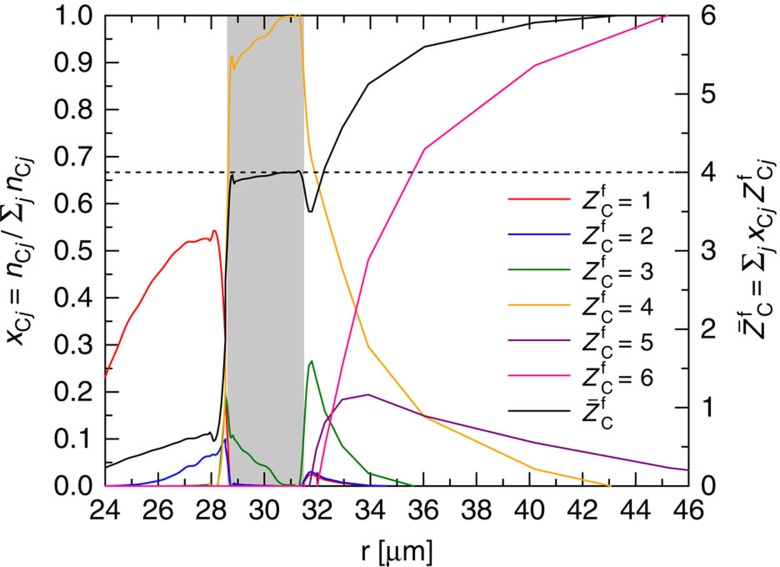
Radial profile of the ionization equilibrium of carbon. Relative populations of carbon charge states in the target based on a degeneracy-corrected Saha–Boltzmann equation^6^. Continuum lowering is self-consistently accounted for according to the Stewart–Pyatt model^35^. The mass density and temperature profiles are given by radiation hydrodynamics simulations at *t*=3.4 ns. The vertical grey-shaded region represents the location of the bulk of the mass in the imploding shell, which dominates the scattering signal.

For these calculations, the mean charge state of carbon is held constant at 
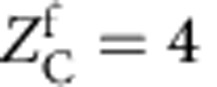
 for all parameters considered. This estimate is supported by previous observations of continuum lowering^21^ based on the shape of the inelastic Compton feature, and also by the excellent agreement we find comparing *ab initio* simulations based on density functional theory (DFT)-molecular dynamics to the predictions of the classical fluid theory for the partial structure factors (see Fig. 1a). Moreover, we have performed self-consistent calculations of the ionization equilibrium^5^ that include continuum lowering^34^ and use temperature and density profiles given by radiation hydrodynamics simulations of the target. Figure 2 clearly shows that the mean charge state in the bulk of the target is dominated by contributions from helium-like carbon.

Before we analyse the data using the screening models, we note that it is possible to obtain the Rayleigh weight directly from the experimental data. This is achieved by decomposing the total scattered power into elastic and inelastic contributions, as per the generalized Chihara formula^26^, and rearranging to obtain


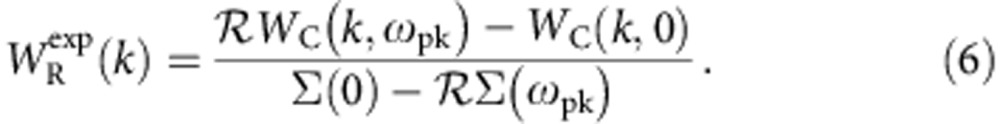


Here, 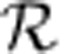 is the ratio of the amplitudes for elastic (*ω*=0) and inelastic (*ω*=*ω*_pk_≈*ħk*^2^/2*m*_*e*_) scattering in the measured spectra, Σ(*ω*) is shape of the X-ray source function and *W*_C_(*k*, *ω*) is the calculated total inelastic contribution to the scattered power.

Using equation (6) to analyse the data, the observed shape and amplitude of the Rayleigh peak is perfectly reproduced. Since it does not depend on any models for the elastic contributions, this approach yields the experimentally determined value for the strength of the Rayleigh peak 
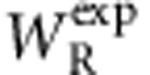
. The resulting conditions of the best fit are then largely determined by the sensitivity of the free-electron response to the density and temperature^19^. Under partially ionized conditions, one may additionally infer the existence or absence of contributions from particular bound states from the shape of the Compton peak; in particular, our data show the absence of L-shell states for the carbon ions^21^.

**Figure 3:**
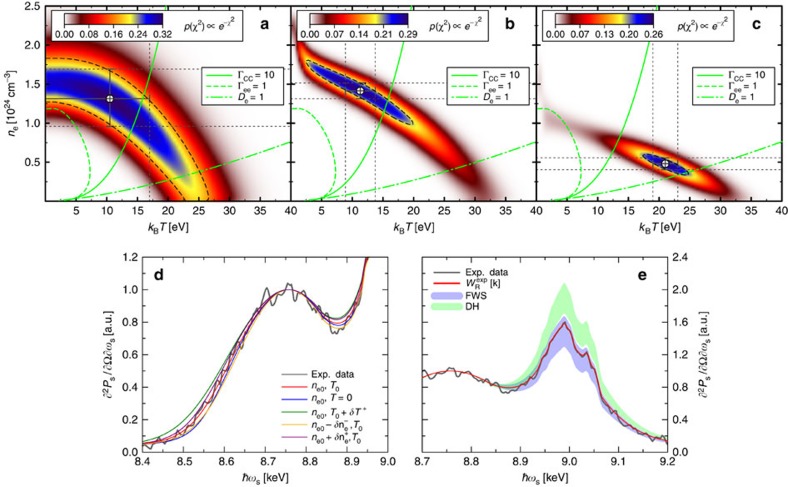
Statistical analysis comparing calculations and experimental data. (**a**–**c**) Colour maps of the probability density function for the *χ*^2^ statistic, *p*(*χ*^2^), comparing experimental data and theoretical calculations. (**a**) Results using the extracted Rayleigh amplitude according to equation (6), (**b**) using the finite-wavelength form given by equations (3) and (4) and (**c**) using the usual Debye-like approach equation (5). The 1*σ* confidence intervals for each plot are marked by the dashed black curves. Contours delineating regimes in which degeneracy and strong coupling become important are further shown by the green curves. Note that Debye-like screening predicted weakly coupled and weakly degenerate states, in contrast to the other approaches. (**d**) Sensitivity of the Compton feature within the errors given by equation (6). (**e**) Comparison of ideal and calculated Rayleigh features at the best fit conditions from **a**, including largest errors at 1*σ*. All panels relate to a pump–probe delay of *t*=3.4 ns.

As demonstrated by Fig. 3, the fitting procedure results in a well-defined state with a clear peak in the probability density. The latter has a highly elongated shape along the temperature axis indicating much greater sensitivity to the electron density, as expected for partially degenerate conditions. For a pump–probe delay of 3.4 ns, the best fit occurs, for example, at *n*_*e*_=1.31(±0.37) × 10^24^ cm^−3^ and *k*_B_*T*=10.5(+6.5) eV. Extracted conditions for delays of 3.5 and 3.6 ns yield decreasing densities and temperatures consistent with cooling and expanding plasmas in the release states after shock compression. This trend agrees with the predictions of radiation hydrodynamics simulations.

The errors on our measurements are quantified by the contour delineating the region in density–temperature space containing 68.3% of the total probability, that is, the 1-sigma confidence interval. Using equation (6) to obtain 
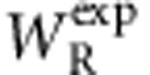
, we find typical error bars of ±20–30% for the density, whereas only an upper limit, typically around 60%, can be rigorously deduced for the temperature due to the temperature insensitivity of the theoretical models for colder, strongly degenerate states. The sensitivity of the inelastic scattering spectra due to such errors is shown in Fig. 3d.

Well-defined conditions are also found when the strength of the Rayleigh feature, *W*_R_, is calculated using the Debye-like model, as well as for the finite-wavelength approaches for the screening cloud (see Fig. 3b,c). We note that in both cases the shape of the contours indicate roughly twice the density sensitivity and substantially improved accuracy with respect to the temperature as compared with the approach determining 
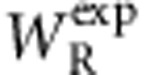
. This stems from the additional constraints due to the temperature and density dependence of the screening cloud. However, the different screening models yield quite different conditions. For example, at the time *t*=3.4 ns, the best fit using finite-wavelength screening is found at *n*_*e*_=1.52(±0.1) × 10^24^ cm^−3^ and *k*_B_*T*=9.3(±2.4) eV, whereas for the Debye-like model, the best fit occurs for *n*_*e*_=4.8(±0.76) × 10^23^ cm^−3^ and *k*_B_*T*=21(±2) eV. Again, cooling and decompression is predicted for later times.

**Figure 4:**
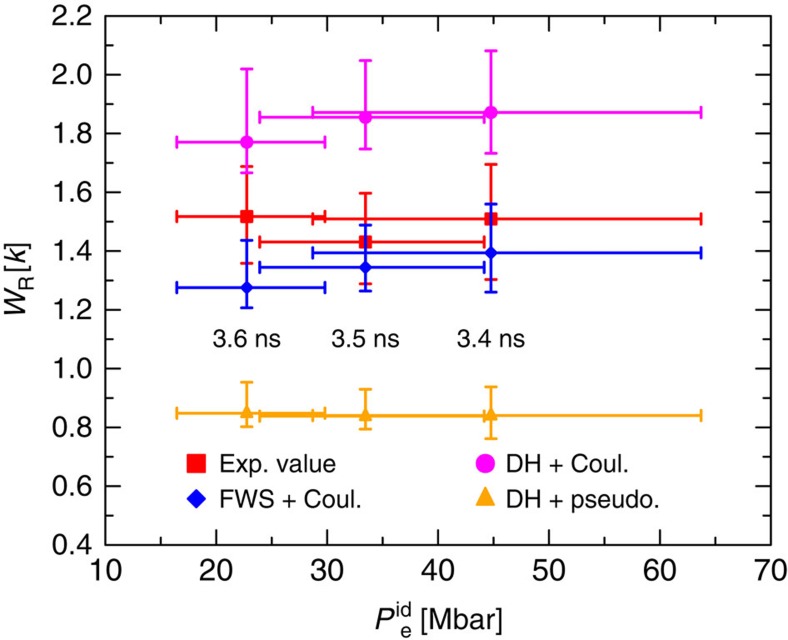
Results for the elastic scattering amplitudes using different models. Rayleigh weight *W*_R_ for various pump–probe delays: extracted values from experimental data (red squares), results using finite wavelength of a Coulomb electron–ion interaction (blue diamonds), Debye–Hückel screening of a Coulomb potential (magenta circles) and a soft-core pseudopotential (orange triangles). The horizontal errors are propagated from the fitting errors based on 
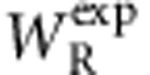
, while the vertical errors also contain contributions from estimated modelling errors, as discussed in the Methods section.

Applied to the same conditions, the elastic scattering strength predicted by different screening models significantly differs. In general, we find that the Rayleigh peak is too large if the *k*→0 (Debye-like) limit is applied, whereas the amplitude given by finite-wavelength screening agrees with the measurements within the error bars (Fig. 3e). Figure 4 compares the elastic Rayleigh strength obtained using the three approaches presented versus the ideal electron pressure 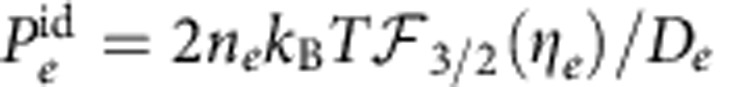 (combining electron density and temperature into a single parameter). Here, we take the plasma conditions obtained from fitting with equation (6) as the reference states, and the corresponding errors are propagated through the calculation of *W*_R_ for each model. We find significant deviations between the results using Debye-like screening and the extracted (experimental) values for all time delays. On the other hand, finite-wavelength screening is in much better agreement for 3.4 and 3.5 ns. For 3.6 ns, the plasma has cooled considerably and one expects strong correlations within the electron gas. The reduction of accuracy found for finite-wavelength screening points to an inadequate description within the RPA as applied here.

Up to now the free electrons have been assumed to respond to the ions via a screened Coulomb-like potential determined by the effective charge of the ions. Clearly, this is justified for the fully ionized hydrogen component. However, the electrons still bound to the carbon ions are known to modify the electron–ion interactions. Here, we estimate this effect using a soft-core pseudopotential similar to the well-known empty-core approach for metals near room temperature^28^. The cutoff radius of the ionic core (*r*_c_=29 pm) is taken from the effective size of C^+4^ ions. The resulting weaker electron–ion interactions lead to oscillations in Fourier space, and negative values of the screening cloud in the range of wavenumbers under study. This effect is exacerbated by the weak decay of the Debye–Hückel/Thomas–Fermi dielectric function in *k*-space. Indeed, within this pseudopotential approach, we do not find a distinct peak in the probability density.

The XRTS data and their analyses clearly show the inapplicability of the long-wavelength approximation inherent to the Debye–Hückel and Thomas–Fermi theories of screening for the high-density matter under investigation. In contrast, the full *k*-dependence of the screening function, which shows a stronger decay for larger wavenumbers and Friedel oscillations in real space near the ion, provides better agreement with measurements under weakly coupled conditions. This finding has important implications when modelling processes with large momentum transfers.

Presently, Debye-like screening builds the basis for theoretical investigations of, for example, the phase diagram^35^, the ion dynamics^36^, the nucleation times^37^ and relaxation properties^10^ of such states. Thus, our understanding of the particle and energy transport in stars or large planets as well as our modelling capabilities for inertial confinement fusion experiments must be improved with respect to better screening models. As the concept of Debye–Hückel-like screening is also used to describe the electron dynamics in solids^38^, quark-gluon matter^6^,^7^ and charged macromolecules^8^, the application of our finding goes far beyond the physics of high-energy-density matter.

## Methods

### Experimental details

The experiments were performed at the Omega Laser Facility at LLE in Rochester, USA, which provides multiple high-energy beams of 351-nm ultraviolet laser light^39^. Eight laser beams with a total energy of 13.5 kJ were used to launch multiple shocks into a 70-μm-thin CH shell. The conditions created by the coalescent shocks were then probed with different pump–probe delays^21^.

Another set of eight laser beams illuminated a Zn foil, creating a hot plasma that emits He-α radiation with a strong peak around 9 keV. These X-rays were used to probe the compressed CH and recorded using a time-gated microchannel plate coupled to a high-resolution highly-oriented pyrolytic graphite (HOPG) crystal spectrometer under scattering angles of 135°±15°. A gold cone protected the spectrometer from direct illumination from the Zn plasma. For non-relativistic probe energies, the momentum transfer from the photons to the plasma, for example, the *k* values probed, is given by the incident X-ray energy *E*_i_ and the scattering angle *θ* via *k*≈(2*E*_i_/*ħc*)sin(*θ*/2) (ref. 13). In the present experiment, we have *k*=8.4–9.2 Å^−1^. In comparison, the inverse screening length in the plasma is *κ*_e_∼2.5 Å^−1^ for the densities and temperatures achieved in the target, yielding *α*=*κ*_e_/*k*∼0.3. Thus, for the relatively large *k* values probed, we expect non-collective scattering and significant contributions from the screening cloud.

### Inelastic scattering contributions

Following the semi-classical approach of Chihara^15^, the dynamic structure factor is decomposed into three different contributions: the scattering from electrons following the ion motion (see equation (2)), scattering from free electrons in the continuum and scattering related to bound-free transitions driven by the incident X-rays^13^. The free-free contribution to the total inelastic scattering is given by the fluctuation-dissipation theorem, which has been evaluated within the RPA^5^.

Considerations beyond the RPA, such as nonlinear coupling between density fluctuations and collisions with the ions, have also been investigated. We estimate strong coupling in the electronic subsystem using static local field corrections^5^ and appeal to the Born–Mermin ansatz^13^ to include screened electron–ion collisions in first Born approximation. We find that nonideal effects beyond RPA yield only minor changes to our fitting procedure. Collisions do not noticeably modify the Compton feature owing to strong Pauli blocking of electron–ion-scattering channels under the degenerate conditions of interest. As shown in Fig. 1b, local field effects do not significantly influence the free-electron response at large *k*.

Finally, bound-free transitions have been treated within the impulse approximation^13^ due to the large *k* values considered and result in a low amplitude tail on the red wing of the inelastic feature. Fortunately, there is only a small overlap between the bound-free and free-free components of the inelastic feature for 
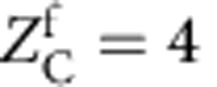
. Thus, the fitting procedure is not strongly sensitive to the bound-free contribution.

### Elastic scattering contributions

Besides the screening function, the modelling of elastic scattering requires the form factor for electrons bound to the carbon ions *f*_C_(*k*) and the partial ion–ion structure factors *S*_*ab*_(*k*). The form factor can be obtained with high precision using DFT calculations, and is also well described (see Fig. 1b) using hydrogenic wavefunctions with tabulated effective screening constants^13^.

The partial static structure factors of the ion subsystem were calculated from both DFT coupled to molecular dynamics^26^ and via a multicomponent generalization of the hypernetted-chain equations from the classical fluid theory^27^. The structure predicted from DFT-molecular dynamics is well reproduced if the ion–ion potential is approximated with a Yukawa-like pseudopotential. We find that finite-wavelength screening of the effective ion–ion interaction is not important, as the inter-ionic separation is sufficiently large at the densities considered for the *k*→0 approximation to be reasonable. Further considerations such as the additional short-range repulsion resulting from the electrons bound to the carbon ions can be included using a simple extension^25^. The latter yields negligible improvements owing to the small size of the effective core radius in helium-like carbon. We find very small deviations from the ideal gas values (unity or zero) for the *k* values probed by our experiment, making our analysis very robust against uncertainties in the ionic structure. Thus, the only significant uncertainty lies in the description of the screening clouds.

### Evaluation of modelling uncertainties

As implied by equation (6), the value of 
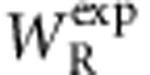
 extracted from the experimental data is sensitive to the shape of the normalized X-ray source function Σ(*ω*). We have considered time-dependent profiles extracted from the data^21^, and also models such as Gaussian, Lorenztian and Voigtian functions. We find that the latter all require stronger elastic scattering to reproduce the amplitude of the observed peak, yielding typical uncertainties of ∼+10%. Moreover, we find that the asymmetric shape of the extracted source function, which implicitly contains the depth-broadening effect of the HOPG crystal^40^, is crucial to simultaneously fitting all spectral features of the data.

The strong angular divergence of the source can be accounted for by averaging calculations over a weighted distribution of scattering angles. Such a consideration has been shown to be important in the collective scattering regime^29^. Here, the scattering is non-collective for all scattering angles considered, and the net effect on the spectrum is a small degree of additional blurring in frequency space. A small impact on the Rayleigh weight stems from enhanced inelastic weight at *ω*=0 and the explicit *k*-dependence of the terms of equation (2). We find that changes to the functional form and width of the weighting distribution yield uncertainties that are well within an accuracy of approximately −5%.

Finally, recent work^41^ for highly compressed plastic targets points to attenuation of the probe X-rays through the target as another potential source of error in *W*_R_. In the present experiment, the much smaller size and lesser degree of compression of the target at the time of measurement ensures that negligible attenuation. Furthermore, radiation hydrodynamics simulations of the implosion suggest that the driven shell of material yields a fairly uniform density distribution in the region that dominates the scattering^21^.

In summary, estimates of error sources have been determined to be on the order of *ɛ*_mod_∼+10%/−5%. These errors are combined with the statistical errors arising from the fitting procedure by summing in quadrature, 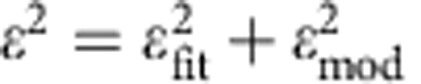.

## Author contributions

D.A.C., J.V., R.A.B. and D.O.G. developed and applied the theory presented. J.V. performed the DFT-MD simulations. L.B.F., L.D., T.D., R.W.F., S.H.G., G.G., A.L.K., T.M. and A.E.P. carried out the experiment and the data were analysed by D.A.C., L.B.F. and T.M.G. D.A.C. and D.O.G. wrote the paper. S.H.G., G.G., O.L.L. and D.O.G. provided additional support for the experiment, data analysis and interpretation.

## Additional information

**How to cite this article:** Chapman, D.A. *et al*. Observation of finite-wavelength screening in high-energy-density matter. *Nat. Commun.* 6:6839 doi: 10.1038/ncomms7839 (2015).
